# Genome-Wide Association Analysis of Reproductive Traits in Chinese Holstein Cattle

**DOI:** 10.3390/genes15010012

**Published:** 2023-12-20

**Authors:** Jiashuang Liu, Lingyang Xu, Xiangbin Ding, Yi Ma

**Affiliations:** 1 Tianjin Key Laboratory of Animal Molecular Breeding and Biotechnology, Tianjin Engineering Research Center of Animal Healthy Farming, Institute of Animal Science and Veterinary, Tianjin Academy of Agricultural Sciences, Tianjin 300381, China; veterinary111@outlook.com; 2 College of Animal Science and Veterinary Medicine, Tianjin Agricultural University, Tianjin 300384, China; xiangbinding@tjau.edu.cn; 3 Institute of Animal Science, Chinese Academy of Agricultural Sciences, Beijing 100193, China; xulingyang@163.com

**Keywords:** genome-wide association study, reproductive traits, Chinese Holstein cattle

## Abstract

This study was to explore potential SNP loci for reproductive traits in Chinese Holstein cattle and identify candidate genes. Genome-wide Association Study based on mixed linear model was performed on 643 Holstein cattle using GeneSeek Bovine 50 K SNP chip. Our results detected forty significant SNP loci after Bonferroni correction. We identified five genes (*VWC2L, STAT1, PPP3CA, LDB3,* and *CTNNA3*) as being associated with pregnancy ratio of young cows, five genes (*PAEP, ACOXL, EPAS1, GLRB,* and *MARVELD1*) as being associated with pregnancy ratio of adult cows, and nine genes (*PDE1B, SLCO1A2, ARHGAP26, ADAM10, APBB1, MON1B, COQ9, CDC42BPB, MARVELD1*, and *HPSE2*) as being associated with daughter pregnancy rate. Our study may provide valuable insights into identifying genes related to reproductive traits and help promote the application of molecular breeding in dairy cows.

## 1. Introduction

Genome-wide Association Study (GWAS) was proposed by Risch and Merikan-gas in 1996 when studying the genetic base of human complex diseases [1]. GWAS applies a Single Nucleotide Polymorphism (SNP) as a molecular genetic marker at genome-wide level and identifies SNPs and candidate genes for target traits [2]. The analysis basis of GWAS is the phenomenon of linkage disequilibrium (LD) caused by recombination in the long-term evolution of natural populations [3]. According to the decay relationship between SNP locus and target trait, the SNP locus can be analyzed using statistics to identify those closely related to phenotypic variation. With the development of sequencing technology and high-throughput genotyping, GWAS has become an important method for analyzing genetic variation. Since the introduction of cattle high-density chips, there are more extensive and in-depth livestock-related GWAS research.

Reproductive traits are important economic traits of dairy cows, including calving interval (CI), calving ease (CE), heifer conception rate (HCR), cow conception rate (CCR), and daughter pregnancy rate (DPR). Among them, HCR, CCR, and DPR can more intuitively represent the reproductive ability of cows. In the past few decades, most intensive selection on milk production traits has led to a decline in the reproductive function of dairy cows, for example, CI, CE, HCR, CCR, and DPR, which indirectly affected the economic benefits [4,5]. Nowadays, the concept of balanced breeding has been popularized and recognized, and the reproductive traits of dairy cows have been given attention. With the continuous maturity of sequencing technology, GWAS studies on the reproductive traits of dairy cows are been reported successively, but there is still a lot of room for research. Olsen et al. [6] performed a GWAS analysis of 2552 Norwegian Red Bull groups from 108 bull families, and target traits mainly included stillbirth, dystocia, and 56-days non-return rate, and found 16 SNPs significantly associated with stillbirth and dystocia. Among them, SNP-rs29012179, located in BTA 12, affects both reproductive and milk production traits. Sahana et al. [7] analyzed on 11 reproductive traits using GWAS and identified 24 QTL regions on 14 chromosomes composed of highly significant SNPs in Danish and Swedish Holstein. Hering et al. [8] performed a GWAS analysis of semen quality with 320 bulls as a test population, finding 34 SNPs that reached genome-level significance. Huang et al. [9] studied the reproductive traits of Holstein cattle using the selective DNA pooling method and found twenty-two SNPs related to fertilization rate and five SNPs related to blastocyst rate. Liu et al. [10]. conducted genome-wide association analysis on the first birth age of HOST cattle and found genes including *KLHL 4 Ls, TRAM 1, TRAM 2, ZNF438* and *MATK* were associated with reproductive disorders within 200 kb upstream and downstream of the significant SNP. Multiple SNP sites within the 1 Mb region of chromosome 7 were significantly associated with the day age of primary labor. However, GWAS analysis of reproductive traits including CI, CE, HCR, CCR, and DPR was not fully explored.

In this study, GWAS was used to explore SNP loci affecting important traits including HCR, CCR and DPR in dairy cows and detect the SNP loci associated with target traits.

## 2. Materials and Methods

### 2.1. Sample Genotype and Quality Control

In this study, the tail root blood was collected from 643 Holstein cows, and 3–5 drops of fresh blood from the tail root of dairy cows were drawn for DNA extraction. The first step involved cell lysis, exposing and rupturing membrane barriers such as cell and nuclear membranes. The next step was to remove membrane lipids from the sample; finally, DNA precipitation involved the removal of DNA-associated proteins by protease and removal of RNAs by RNase. The extracted DNA samples were quantitatively detected by a microspectrophotometer, adding DNA samples directly to the base, using two optical fibers to form the detection path, and with full-spectrum detection, the OD value of the DNA samples could be obtained. The DNA genome of Holstein cattle was scanned and analyzed using the Genomic Profiler Bovine 100 K chip containing 88,107 SNPs. Final genotyping data were obtained for the SNP. Quality control of individual data and SNP data in PLINK (v1.9) [11], began by first converting the SNP chip data into files that could be read by the PLINK (v1.9) software (*.map and *.Pad). The converted files included: individual ID, male parent ID (0 without record), female parent ID (0 without record), sex, SNP allele, chromosome, physical location, and genetic distance. A total of 637 individuals and 88,107 SNPs were obtained. The quality control conditions were as follows: (1) individual data with SNP genotype missing rates greater than 10% were filtered out; (2) SNP data with detection rate less than 90% were filtered out; (3) SNP data with a minimum allele frequency of less than 5% were removed; and (4) SNP data with Hardy-Weinberg equilibrium test *P* values less than 1.0 × 10^−7^ were removed.

After quality control, a total of 76,160 high-quality SNPs from 637 samples were used for subsequent GWAS analysis. The quality control command in PLINK software were as follows: PLINK- -bfile filename- -mind 0.1- -geno 0.1- -maf 0.05- -hwe 0.0000001- -make-bed- -out outfilename. The output file contained the individual data information and the SNP data information after quality control (Table 1). Information on the distribution of SNP markers on each chromosome before and after quality control is shown in Table 2.

### 2.2. Statistical Analysis

Principal component analysis (PCA) was evaluated with PLINK (v1.9). The population was corrected for PCA results, thus avoiding false positives [12] due to population stratification. GWAS analysis between reproductive traits and genome-wide SNPs is performed using a mixed linear model in the GEMMA (v0.98.5) software [13], establishing the model as follows:

y = μ + Xβ + Zv + e

In the formula: y is a vector of n × 1, for breeding values after removing genomic effects; μ represents the mean value of the trait phenotypic values; X is the mixed-effect matrix of n × q; β is a vector of q × 1, for the coefficient of the fixed effect; Z is the random polygene effect relationship matrix of n × t; v is the vector influenced by many factors, following the distribution of N (0, K$\sigma_{\alpha }^{2}$), where K is related to all SNP genotypes and $\sigma_{\alpha }^{2}$ additive variance, and is the relatedness matrix of t × t; e is the residual vector following the N (0, *I*$\sigma_{\alpha }^{2}$) distribution about the unknown variance $\sigma_{\alpha }^{2}$.

### 2.3. Significance Test

Bonferroni correction was conducted for multiple tests. The significance level of the single test is: chromosome level (1/*n*), genome-wide level (0.05/*n*), where *n* is the number of SNP after quality control. Therefore, the chromosome level significant threshold is (1/76160 = 1.31 × 10^−5^), and the genome-wide level significant threshold is (0.05/76160 = 6.57 × 10^−7^).

### 2.4. Population Stratification Analysis

To determine whether the population stratification affected the association between SNPs and traits, Q-Q plots (Quantile-Quantile plot) were drawn using R v3.5.1 [14].

### 2.5. Screening and Annotation of the Candidate Genes

Based on National Animal Genome Research Program and National Center for Biotechnology databases, ARS-UCD 1.2 was used as the reference genome. Candidate genes for HCR, CCR, and DPR were searched within 50 kb the upstream or downstream of significant SNPs, and genes functions were annotated [15].

### 2.6. Enrichment Analysis of the Candidate Genes

The enrichment analysis of reproductive trait-related candidates obtained by GWAS analysis was conducted with the DAVID database, and identified the pathways in which these genes were involved.

### 2.7. Software and the Database Website Used in This Study

PLINK (v1.9): http://pngu.mgh.harvard.edu/~purcell/PLINK/ (accessed on 3 February 2023).

GEMMA (v0.98.5): https://github.com/genetics-statistics/GEMMA (accessed on 15 March 2023).

R (3.5.1): https://cran.r-project.org/mirrors.html (accessed on 18 March 2023).

The UCSC database: http://genome.ucsc.edu/ (accessed on 6 April 2023).

The NCBI database: http://www.ncbi.nlm.nih.gov/ (accessed on 6 April 2023).

ENSEMBL database: http://asia.ensembl.org/index.html (accessed on 11 May 2023).

Animal QTL database: http://www.animalgenome.org/cgi-bin/QTLdb/index/ (accessed on 6 April 2023).

The DAVID database: http://david.ncifcrf.gov/list.jsp (accessed on 26 August 2023).

The KEGG database: https://www.kegg.jp/ (accessed on 26 August 2023).

## 3. Results

### 3.1. Population Stratification

**Figure 1:**
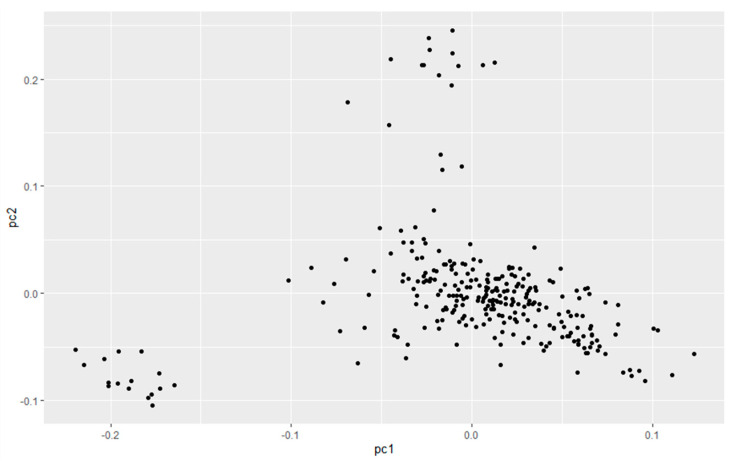
Principal component analysis of 643 Chinese Holstein cattle.

**Figure 2:**
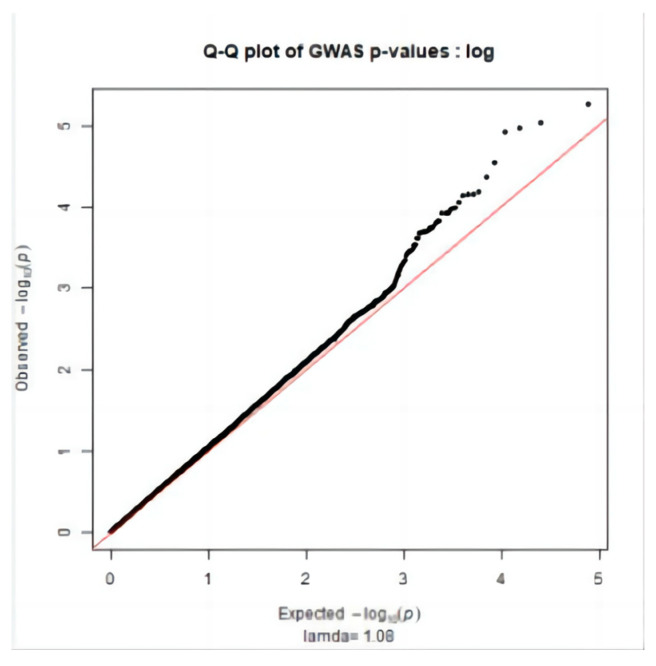
HCR Q-Q Figure.

**Figure 3:**
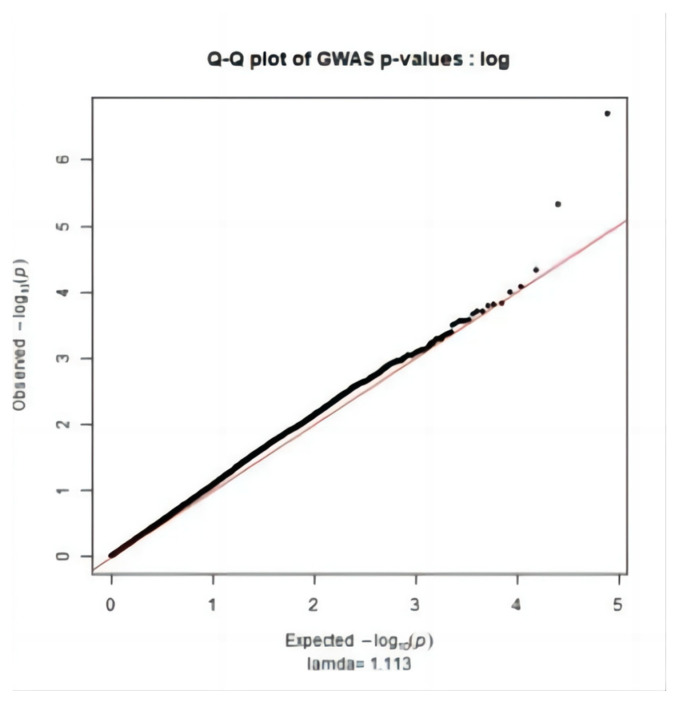
CCR Q-Q Figure.

**Figure 4:**
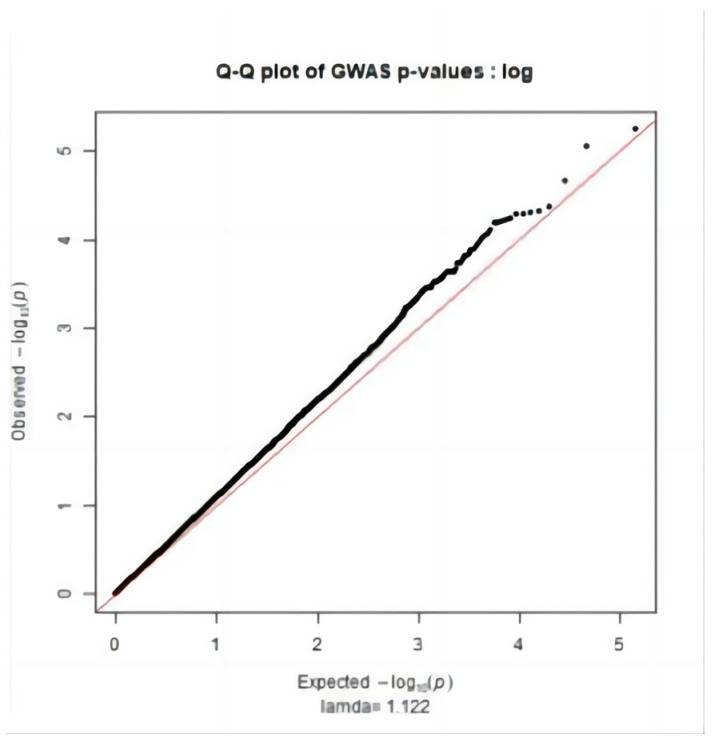
DPR Q-Q Figure.

Figure 1 main component analysis revealed the sample population was divided into groups (PC1: 0~−1.5, PC2: 0~−0.05; PC1: −0.05~0, PC2: 0.2~0.25; PC1: −0.1~0.1, PC2: −0.1~0.05). Therefore, when performing association analysis on the whole genome after quality control, using population relatedness as a random effect relationship matrix in the mixed linear model can avoid false positive [16,17,18] of results due to population stratification. The Q-Q diagram was drawn with R (3.5.1), as shown in Figure 2, Figure 3 and Figure 4. The ordinate is −log_10_ (GWAS *p*-value observations), the abscissa is −log_10_ (GWAS *p*-value predictions), and the red line is straight line Y = X. It can be seen the distribution of the χ^2^ statistic calculated by the SNP association analysis does not deviate prematurely from the hypothesis test, indicating the analytical model used complies with this study. A deviation occurs on the upper right side of the figure, indicating SNP sites [19] are significantly correlated with HCR, CCR, and DPR, respectively.

### 3.2. Genome-Wide Association Study

**Figure 5:**
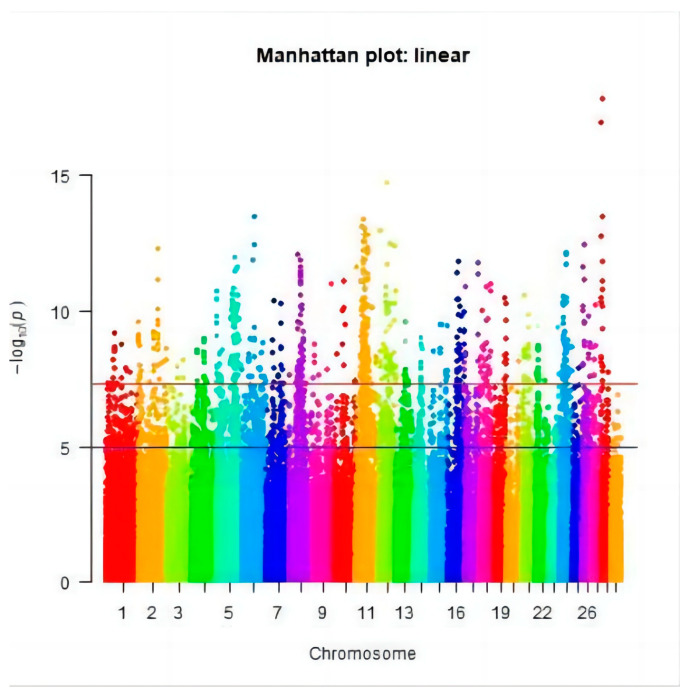
The Manhattan plot of the HCR.

**Figure 6:**
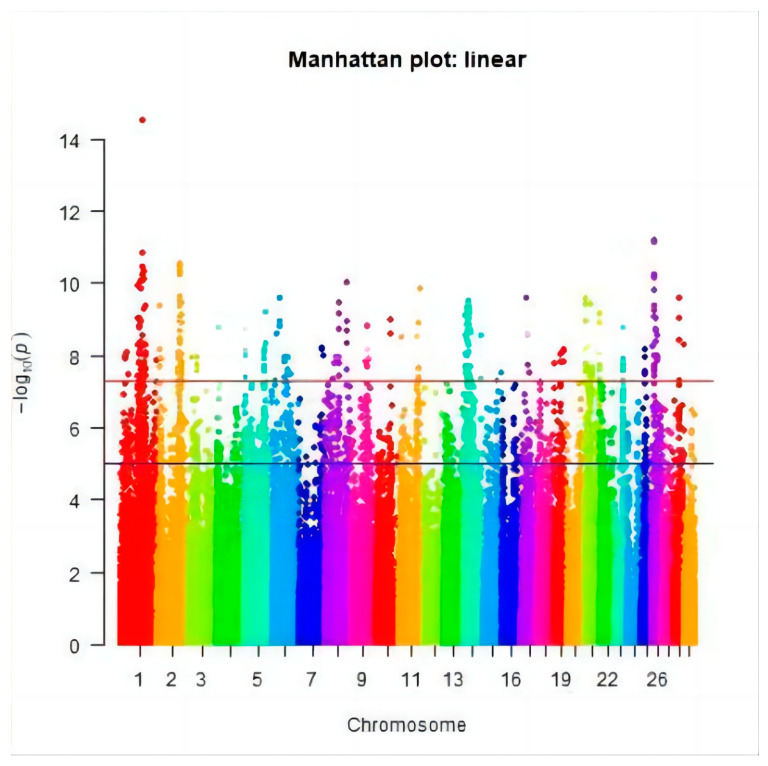
The Manhattan plot of the CCR.

**Figure 7:**
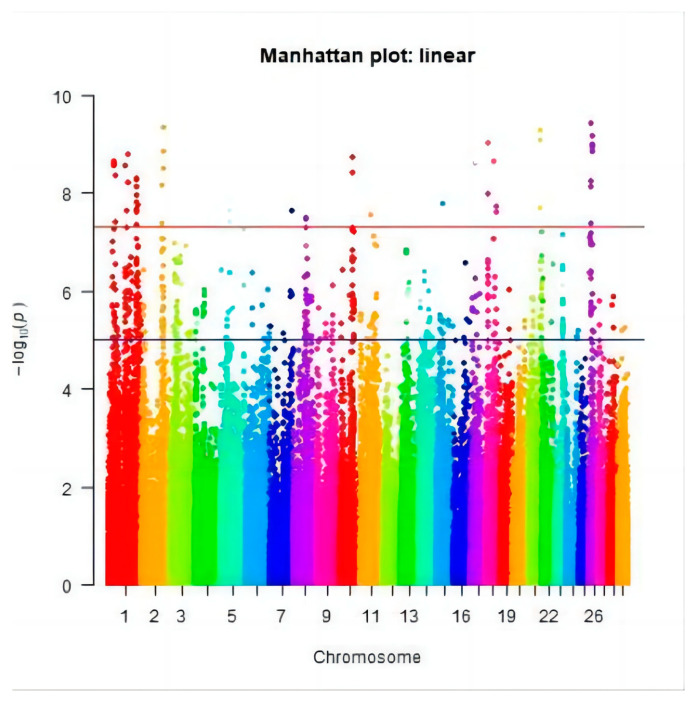
The Manhattan plot of the DPR.

After quality control, the genotypes of 88,107 SNPs were obtained in 637 cows. Genome-wide association analysis was conducted on HCR, CCR, and DPR in Chinese Holstein cattle, and a Manhattan plot was generated as shown in Figure 5, Figure 6 and Figure 7 (different colors represent the different chromosome numbers). After Bonferroni correction, eight SNPs were significantly associated with HCR at the chromosome level (*P* < 1/76160), eight SNPs were significantly associated with CCR at the chromosome level (*P* < 1/76160), and twenty-four SNPs were significantly associated with DPR at the chromosome level (*P* < 1/76160).

### 3.3. Significant SNPs and the Candidate Genes

After Bonferroni correction, 40 SNPs were found to be supported by statistical significance: eight SNPs significantly related to HCR were detected on chromosomes 2, 6, 12, and 28 (Figure 5); eight SNPs significantly connected to CCR were located on chromosomes 1, 8, 11, 17, and 26 (Figure 6); and twenty-four SNPs loci significantly associated with DPR were located on chromosomes 2, 5, 7, 10, 15, 18, 21, and 26 (Figure 7). These 40 significant SNPs were annotated, and the 20 closest candidate loci were obtained (Table 3).

### 3.4. Enrichment Analysis of the Candidate Genes

**Figure 8:**
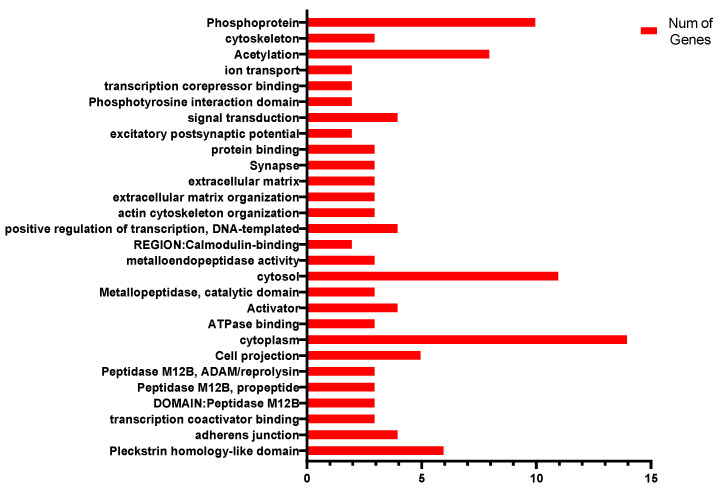
GO enrichment results for candidate genes for reproductive traits.

GO enrichment of reproductive trait candidate genes using DAVID database found 28 biological reactions were associated in vivo, each involving zero to fifteen genes (Figure 8). The KEGG analysis revealed no pathway where the candidate genes were located.

## 4. Discussion

In this study, we performed a genome-wide association analysis based on a mixed linear model of reproductive-related traits for a population using the GeneSeek Genomic Profiler Bovine 50 K SNP chip in 643 Chinese Holstein cattle. The PLINK software was used to perform the quality control and principal component analysis of the individual and genotype data. The principal component analysis found the sample population could be divided into three groups, which had an indicative role in avoiding the emergence of false positives and the selection of models due to population stratification [20]. In the mixed linear model, SNP effects and population stratification effects were used as fixed effects and kinship matrix as random effects for subsequent GWAS analysis, which greatly improved the accuracy of the association analysis [21,22].

### 4.1. Genome-Wide Association Study

We identified candidate genes for production traits. Candidate genes were identified by the NCBI and other databases; five candidate genes were associated with HCR: von Willebrand factor C domain containing 2 like (*VWC2L*), signal transducer and activator of transcription 1 (*STAT1*), protein phosphatase 3 catalytic subunit α (*PPP3CA*), LIM domain binding 3 (*LDB3*), and catenin α 3 (*CTNNA3*); five candidate genes associated with CCR: progestagen associated endometrial protein (*PAEP*), acyl-CoA oxidase like (*ACOXL*), endothelial PAS domain protein 1 (*EPAS1*), glycine receptor β (*GLRB*), and MARVEL domain containing 1 (*MARVELD1*); and ten candidate genes were associated with DPR: phosphodiesterase 1B (*PDE1B*), solute carrier organic anion transporter family member 1A2 (*SLCO1A2*), Rho GTPase activating protein 26 (*ARHGAP26*), ADAM metallopeptidase domain 10 (*ADAM10*), amyloid β precursor protein binding family B member 1 (*APBB1*), MON1 homolog B, secretory trafficking associated (*MON1B*), coenzyme Q9 (*COQ9*), CDC42 binding protein kinase β (*CDC42BPB*), MARVEL domain containing 1 (*MARVELD1*), and heparanase 2 (*HPSE2*).

#### 4.1.1. Candidate Genes Associated with the HCR

*VWC2L* is located on chromosome 2 in dairy cows. *VWC2L* was previously identified as a member of the cysteine desmin (CKP) family. A previous study suggests it is a novel secreted protein that regulates the function of osteoblast cells and promotes matrix mineralization by regulating the Osterix expression of TGF-βsuperfamily growth factor signaling pathway [23]. Wang Kejun et al. [24] found *VWC2L* gene was significantly associated with food conversion rate, involved in the growth metabolism, and indirectly associated with the growth traits of pigs. Therefore, *VWC2L* promotes the growth and metabolism of Holstein cattle, and it promotes the pregnancy of young cows by accelerating the formation of fetal bone.

*STAT1* was detected on chromosome 2. Signal transducers and activators of transcription (STAT) proteins are essential for the regulation of many biological processes. In cattle, microarray analysis identified *STAT1* as differentially expressed genes in the implanted surrounding endometrium. To gain new insights regarding *STAT1* during the estrous cycle and early pregnancy, Carvalho et al. [25] investigated *STAT1* transcript and protein expression and its biological activity in bovine tissue and endometrial-derived cells. This study found pregnancy increased *STAT1* expression on day 16, *STAT1* was located in endometrial cells on day 20 of pregnancy, and increased binding of *STAT1* to interferon regulatory factor 1 (*IRF1*), cytokine-induced SH2-containing protein (CISH), and cytokine signaling 1 and 3 (*SOCS1, SOCS3*) gene promoters was consistent with the induction of its transcripts. The high expression of *STAT1* in endometrial cells during pregnancy confirmed its association with pregnancy in young cows.

The *PPP3CA* gene, a candidate gene for goat high reproductive traits revealed by genome-wide association study, is located on chromosome 6 in cattle and is significantly associated with the pregnancy matching rate in young cows. Yangyang et al. [26] explored the genetic variation of goat *PPP3CA* and evaluated the genetic impact on litter size. They found only a 20bp insertion-deletion polymorphism in northern Shaanxi white cashmere goats was significantly associated with litter number (*p* < 0.05), and individuals with deletion/deletion (DD) genotype showed a primary phenotype compared with individuals with other genotypes. This study demonstrated deletion mutations within the *PPP3CA* gene in goats significantly affected litter size. By UniProt comparison, the *PPP3CA* protein of Holstein cattle and goats are as high as 98.40%, and the *PPP3CA* gene, as a candidate gene for the pregnancy rate of young cows, may have some influence on the reproductive traits of Holstein cattle population.

*LDB3*, located on bovine chromosome 28, is a well-characterized striated PDZ-LIM protein that regulates mechanical stress signaling [27] by association with the mechanosensing domain in filamin C. *CTNNA3,* also located on bovine chromosome 28, is implicated with preferential expression of the maternal allele in early gestational placental tissue [28] and differentiated villous or extravillous trophoblast. Whether these two genes can be used as candidate genes in young cows needs further confirmation.

#### 4.1.2. Candidate Genes Associated with the CCR

*PAEP* is located on chromosome 11 in cattle. *PAEP* is a progesterone-associated endometrial protein, and studies demonstrate this gene enhances cell proliferation through the receptor-mediated membrane IgM receptor, and is the main component in regulating cell proliferation in milk [29]. As a 28-kD glycoprotein, *PAEP* constitutively expresses in both the human reproductive tract and the hematopoietic system [30]. Therefore, *PAEP* promotes cell proliferation under the action of progesterone, which has a positive impact on the pregnancy matching rate in adult cows.

*ACOXL* is located on chromosome 11. Ana Paula Sbardella et al. [31] used genome-wide association analysis to study reproductive traits in Nelore cattle and found *ACOXL* was significantly associated with multiple reproductive traits, and this gene could also serve as a candidate gene affecting reproductive traits in Chinese Holstein cattle.

*MARVELD1* is located on chromosome 26 of dairy cows, which is associated with CCR and DPR. By gene mapping of the mouse cell cycle, Zeng Fanli et al. [32] found overexpression of mouse *MARVELD1* resulted in significant inhibition of cell proliferation, G1 arrest, and reduced cell migration. This study showed *MARVELD1* was a microtubule-associated protein that played an important role in cell cycle progression and migration and had an effect on adult cows and daughter pregnancies.

*EPAS1* is located on chromosome 11 in cattle and plays multiple supporting roles in maintaining specific aspects of adipocyte function, including regulation of glucose uptake and lipid synthesis [33]. *GLRB* (glycine receptor β), located on chromosome 17, is a regulator of motor neuron numbers [34]. These two genes may be candidates for the pregnancy rate in adult cows and require experimental validation.

#### 4.1.3. Candidate Genes Associated with the DPR

*PDE1B* is located on bovine chromosome 5. Previous studies have shown the *PDE1B* gene could be regarded as a positional and functional candidate gene for carcass traits in beef cattle. Zhou Di et al. [35] used high-throughput sequencing to analyze the *MyoD1* gene knockout transcriptome of MDBK cells (bovine kidney cells) and found *PDE1B* was highly expressed in the vine leg muscle, dorsal long muscle, and shoulder, which was related to muscle formation and differentiation. Therefore, *PDE1B* has a certain promoting effect on fetal muscle formation and differentiation, and it has a positive effect on daughter pregnancy, and *PDE1B* can be further identified as a candidate gene for DPR.

*SLCO1A2* is located on bovine chromosome 5; it is a member of the solute carrier organic anion transporter family. Joachim Geyer et al. [36] encode two different full-length *SLCO1A2* cDNA as 666 amino acid membrane proteins containing 12 putative transmembrane-spanning domains. Bovine *SLCO1A2* expression was detected in the liver, kidney, brain, and adrenal glands. This study also confirmed bovine *SLCO1A2* had functional homology with human *SLCO1A2*, which had a certain role in the growth and development of various fetal organs, and could be further verified as a candidate gene for DPR.

*ADAM10* is located on bovine chromosome 10. It is found *ADAM10* initiates the hydrolysis of regulatory membrane proteins by shedding the ectodomains of many different substrates, participates in cell-cell interaction and cell migration [37], has a certain role in the functional maintenance of organs and tissues during pregnancy, and can be further identified as a candidate gene related to DPR.

*APBB1* is located on bovine chromosome 15. APBB1 is described as a bridging protein, acting in concert with the β- amyloid precursor protein (APP) and Tip60 (histone acetyltransferase). Studies have shown *APBB1* played a dominant role in nuclear signaling [38]. This gene could be further identified as a candidate gene for DPR.

*MON1B* is located on bovine chromosome 18. It has been shown *MON1B* participated in blastocyst formation to promote embryonic development, and gene expression [39] existed in mature oocytes and embryos.

*COQ9* is located on bovine chromosome 18. To explore the effects of single nucleotide polymorphisms in *COQ9* on mitochondrial, ovarian function, and fertility in Holstein cows, M Sofia Ortega et al. [39] evaluated fertility phenotype measures of up to five lactation in a population of 2273 Holstein cows and showed the coenzyme Q9 mutation affected ovarian function. The A allele was associated with increased mitochondrial DNA copy number in oocytes, with overdominance effects on oocyte *COQ9* expression, follicle number, and anti-mullerian hormone concentration. *COQ9* is associated with fertility in Holstein cattle and can be a candidate gene influencing DPR.

*ARHGAP26, CDC42BPB, HPSE2* are located on chromosomes 7, 21, and 26, respectively. The mechanism of these three genes related to pregnancy is not clear, and its effect on the pregnancy rate needs to be further explored.

### 4.2. Candidate-Gene Enrichment Analysis

GO term function enrichment and KEGG pathway enrichment for the overall different genes, do not distinguish which different gene is up- or down-regulated, and cannot reflect the overall form of activation or inhibition. However, these analyses can categorize genes according to the cell components, molecular function, and biological processes following a multiple gene set analysis, so that researchers can better understand gene function. KEGG pathway integration of the information of genomics, biochemistry, and system functional omics helps researchers to study gene and expression information as a whole, to better understand the mechanism of gene regulation, and also to judge whether the candidate genes of each trait are reasonable, and provide reference for subsequent identification tests.

In this study, GO enrichment found 28 biological reactions were correlated with reproductive traits, with each reaction involving zero to fifteen genes. The specific regulatory mechanism needs to be explored.

## 5. Conclusions

In this study, genome-wide association analysis of reproductive traits in 643 Chinese Holstein cattle population in Tianjin, based on a mixed linear model, identified multiple SNP loci significantly associated with the target trait and identified corresponding candidate genes, but most of the genes were not confirmed and a further identification is needed. This study provides the theoretical basis and breeding direction for the improvement of the breeding performance of Chinese Holstein cattle, and also lays a foundation for further mining and identifying the important gene loci of dairy cows in Tianjin, improving the molecular breeding theory and improving the production performance of dairy cows.

## Figures and Tables

**Table 1:** Descriptive statistics for reproductive traits.

Trait	Number	Average	Maximum	Minimum	Variance	Standard Deviation
HCR	637	0.81	4.1	−2.4	1.30	1.14
CCR	637	0.62	4.9	−3.1	1.69	1.30
DPR	637	0.00031	3.4	−3.2	1.27	1.13

**Table 2:** Information on the distribution of SNP markers on each chromosome before and after quality control.

		Before Quality Control		After Quality Control	
Chromosome	Length (Mb)	SNPs Count	Density (kb/snp)	SNPs Count	Density (kb/snp)
1	158.3	5556	35.1	4693	29.6
2	137.1	4688	34.2	3917	28.6
3	121.4	4508	37.1	3749	30.9
4	120.8	4049	33.5	3390	28.1
5	121.2	4523	37.3	3734	30.8
6	119.5	4364	36.5	3617	30.3
7	112.6	3903	34.7	3232	28.7
8	113.4	3805	33.6	3289	29.0
9	105.7	3695	35.0	3181	30.1
10	104.3	3626	34.8	3116	29.9
11	107.3	3801	35.4	3216	30.0
12	91.2	3044	33.4	2563	28.1
13	84.2	3064	36.4	2617	31.1
14	84.6	3045	36.0	2561	30.3
15	85.3	3119	36.6	2674	31.3
16	81.7	2826	34.6	2338	28.6
17	75.2	2668	35.5	2286	30.4
18	66.0	2605	39.5	2197	33.3
19	64.1	2726	42.5	2226	34.7
20	72.0	2737	38.0	2253	31.3
21	71.6	2573	35.9	2167	30.3
22	61.4	2201	35.8	1878	30.6
23	52.5	2110	40.2	1774	33.4
24	62.7	2259	36.0	1934	30.8
25	42.9	1726	40.2	1443	33.6
26	51.7	1823	35.3	1551	30.0
27	45.4	1699	37.4	1488	32.8
28	46.3	1735	37.5	1506	32.5
29	51.5	1871	36.3	1570	30.5

**Table 3:** Results of genome-wide association analysis of reproductive traits in Chinese Holstein cattle.

Character	SNP	Chromosome	Site (bp)	*p* Value	The Gene Region	Distance (bp)
*HCR*	BovineHD0200029628	2	102,699,078	9.18 × 10^−3^	VWC2L	81,620
	BovineHD0200022967	2	79,537,846	3.11 × 10^−5^	STAT1	21,534
	BTA-37416-no-rs	6	23,807,252	8.69 × 10^−4^	PPP3CA	36,965
	BovineHD28000743	28	41,387,894	7.36 × 10^−5^	LDB3	2984
	BovineHD2800005891	28	22,263,728	6.23 × 10^−5^	CTNNA3	10,962
*CCR*	LGB_X14710_5174	11	103,259,143	7.68 × 10^−3^	PAEP	1719
	BovineHD1100000394	11	1,230,012	3.72 × 10^−4^	ACOXL	6654
	BovineHD4100008487	11	2,827,428	2.86 × 10^−4^	EPAS1	73,158
	BovineHD1700011952	17	42,103,930	4.12 × 10^−4^	GLRB	6465
	BovineHD2600004843	26	18,941,126	4.61 × 10^−5^	MARVELD1	68,791
*DPR*	ARS-USMARC-666	5	25,527,614	1.51 × 10^−6^	PDE1B	11,893
	BovineHD0500025349	5	88,911,724	6.17 × 10^−4^	SLCO1A2	22,340
	BovineHD0700016212	7	54,281,313	6.66 × 10^−4^	ARHGAP26	3543
	Hapmap58695-rs29019899	10	51,673,875	1.95 × 10^−5^	ADAM10	5385
	BovineHD1500013531	15	46,637,606	1.29 × 10^−5^	APBB1	11,182
	UFL-rs41859871	18	4,419,224	1.94 × 10^−5^	MON1B	3866
	COQ9_rs109301586	18	25,446,323	4.27 × 10^−4^	COQ9	2139
	ARS-BFGL-NGS-115062	21	67,739,884	8.39 × 10^−3^	CDC42BPB	18,754
	ARS-BFGL-NGS-115980	26	20,196,747	1.74 × 10^−5^	HPSE2	61,344
	BovineHD2600004843	26	18,941,126	4.61 × 10^−5^	MARVELD1	68,791

## Data Availability

No new data were created or analyzed in this study. Data sharing is not applicable to this article.

## References

[1] Risch N., Merikangas K. (1996). The Future of Genetic Studies of Complex Human Diseases. Science.

[2] Iniguez_Luy H.A.G.B. (2015). Association mapping of seed quality traits inBrassica napusl using GWAS and candidate QTL approaches. Mol. Breed..

[3] Hirschhorn J.N., Daly M.J. (2005). Genome-wide association studies for common diseases and complex traits. Nat. Rev. Genet..

[4] Hermas SA Young C.W., Rust J.W. (1987). Genetic Relationships and Additive Genetic Variation of Productive and Reproductive Traits in Guernsey Dairy Cattle. J. Dairy Sci..

[5] Ali I., Muhammad Suhail S., Shafiq M. (2019). Heritability estimates and genetic correlations of various production and reproductive traits of different grades of dairy cattle realed under subtropical condition. Reprod. Domest. Anim..

[6] Olsen H.G., Hayes B.J., Kent M.P., Nome T., Svendsen M., Lien S. (2010). A genome wide association study for QTL affecting direct and maternal effects of stillbirth and dystocia in cattle. Anim. Genet..

[7] Sahana G., Guldbrandtsen B., Thomsen B., Lund M.S. (2013). Confirmation and fine-mapping of clinical mastitis and somatic cell score QTL in Nordic Holstein cattle. Anim. Genet..

[8] Hering D., Olenski K., Kaminski S. (2014). Genome-wide association study for poor sperm motility in Holstein-Friesian bulls. Anim. Reprod. Sci..

[9] Huang W., Kirkpatrick B.W., Rosa G.J.M., Khatib H. (2010). A genome-wide association study using selective DNA pooling identifies candidate markers for fertility in Holstein cattle. Anim. Genet..

[10] Liu A., Guo G., Wang Y. (2015). Genetic assessment and genome-wide association analysis of Holstein cattle in China. J. Anim. Husb. Vet. Med..

[11] Chang C.C., Chow C.C., Tellier L.C., Vattikuti S., Purcell S.M., Lee J.J. (2015). Second-generation PLINK: Rising to the challenge of larger and richer datasets. GigaScience.

[12] Yang J., Weedon M.N., Purcell S., Lettre G., Estrada K., Willer C.J., Visscher P.M. (2011). Genomic inflation factors under polygenic inheritance. Eur. J. Hum. Genet..

[13] Zhou X., Stephens M. (2012). Genome-wide efficient mixed-model analysis for association studies. Nat. Genet..

[14] Jiang L., Liu J., Sun D., Ma P., Ding X., Yu Y., Zhang Q. (2010). Genome Wide Association Studies for Milk Production Traits in Chinese Holstein Population. PLoS ONE.

[15] Wang T., Gao X., Song W., Yao D., Chen L., Chen C., Ma Y. (2021). Genome-wide association analysis of limb-hoof structure and breast morphology in Chinese Holstein cattle. J. Livest. Ecol..

[16] Pritchard J.K., Stephens M., Rosenberg N.A., Donnelly P. (2000). Association Mapping in Structured Populations. Am. J. Hum. Genet..

[17] Wang W.Y.S., Barratt B.J., Clayton D.G., Todd J.A. (2005). Genome-wide association studies: Theoretical and practical concerns. Nat. Rev. Genet..

[18] Ioannidis J.P.A. (2005). Why Most Published Research Findings Are False. PLoS Med..

[19] Li Y. (2020). Genome-Wide Association Analysis of Biochemical Components of Blood in Chinese Holstein Dairy Cows. Master’s Thesis.

[20] Vilhjálmsson B.J., Nordborg M. (2013). The nature of confounding in genome-wide association studies. Nat. Rev. Genet..

[21] Kim S., Xing E.P. (2009). Statistical estimation of correlated genome associations to a quantitative traits network. PLoS Genet..

[22] O’Reilly P.F., Hoggart C.J., Pomyen Y., Calboli F.C., Elliott P., Jarvelin M.R., Coin L.J. (2012). Multi-Phen: Joint model of multiple phenotypes can increase discovery in GWAS. PLoS ONE.

[23] Ohyama Y., Katafuchi M., Almehmadi A., Venkitapathi S., Jaha H., Ehrenman J., Morcos J., Aljamaan R., Mochida Y. (2012). Modulation of matrix mineralization by Vwc2-like protein and its novel splicing isoforms. Biochem. Biophys. Res. Commun..

[24] Wang K., Liu D., Hernandez-Sanchez J., Chen J., Liu C., Wu Z., Fang M., Li N. (2015). Genome Wide Association Analysis Reveals New Production Trait Genes in a Male Duroc Population. PLoS ONE.

[25] Carvalho A.V., Eozenou C., Healey G.D., Forde N., Reinaud P., Chebrout M., Gall L., Rodde N., Padilla A.L., Delville C.G. (2016). Analysis of STAT1 expression and biological activity reveals interferon-tau-dependent STAT1-regulated SOCS genes in the bovine endometrium. Reprod. Fertil. Dev..

[26] Bai Y., Li J., Zhu H., Liu J., Dong S., Li L., Qu L., Chen H., Song X., Lan X. (2021). Deletion mutation within the goat PPP3CA gene identified by GWAS significantly affects litter size. Reprod. Fertil. Dev..

[27] Pathak P., Blech-Hermoni Y., Subedi K., Mpamugo J., Obeng-Nyarko C., Ohman R., Molloy I., Kates M., Hale J., Stauffer S. (2021). Myopathy associated LDB3 mutation causes Z-disc disassembly and protein aggregation through PKCα and TSC2-mTOR downregulation. Commun. Biol..

[28] van Dijk M., Mulders J., Könst A., Janssens B., van Roy F., Blankenstein M., Oudejans C. (2004). Differential downregulation of αT-catenin expression in placenta: Trophoblast cell type-dependent imprinting of the CTNNA3 gene. Gene Expr. Patterns.

[29] Tai C.S., Chen Y.Y., Chen W.L. (2016). *β*-Lactoglobulin Influences Human Immunity and Promotes Cell Proliferation. BioMed Res. Int..

[30] Kämäräinen M., Riittinen L., Seppälä M., Palotie A., Andersson L.C. (1994). Progesterone-associated endometrial protein--a constitutive marker of human erythroid precursors. Blood.

[31] Sbardella A.P., Watanabe R.N., da Costa R.M., Bernardes P.A., Braga L.G., Baldi Rey F.S., Lôbo R.B., Munari D.P. (2021). Genome-Wide Association Study Provides Insights into Important Genes for Reproductive Traits in Nelore Cattle. Animals.

[32] Zeng F., Tian Y., Shi S., Wu Q., Liu S., Zheng H., Yue L., Li Y. (2011). Identification of mouse MARVELD1 as a microtubule associated protein that inhibits cell cycle progression and migration. Mol. Cells.

[33] Shimba S., Wada T., Hara S., Tezuka M. (2004). EPAS1 Promotes Adipose Differentiation in 3T3-L1 Cells. J. Biol. Chem..

[34] Baguma-Nibasheka M., Fracassi A., Costain W.J., Moreno S., Kablar B. (2016). Role of skeletal muscle in motor neuron development. Histol. Histopathol..

[35] Zhou D., Wang Y., Yang R., Wang F., Zhao Z., Wang X., Xie L., Tian X., Wang G., Li B. (2022). The *MyoD1* Promoted Muscle Differentiation and Generation by Activating *CCND2* in Guanling Cattle. Animals.

[36] Geyer J., Döring B., Failing K., Petzinger E. (2004). Molecular cloning and functional characterization of the bovine (Bos taurus) organic anion transporting polypeptide Oatp1a2 (Slco1a2). Comp. Biochem. Physiol. Part B Biochem. Mol. Biol..

[37] Wild-Bode C., Fellerer K., Kugler J., Haass C., Capell A. (2006). A Basolateral Sorting Signal Directs ADAM10 to Adherens Junctions and Is Required for Its Function in Cell Migration. J. Biol. Chem..

[38] Yang Z., Cool B.H., Martin G.M., Hu Q. (2006). A Dominant Role for FE65 (APBB1) in Nuclear Signaling. J. Biol. Chem..

[39] Ortega M.S., Wohlgemuth S., Tribulo P., Siqueira L.G.B., Null D.J., Cole J.B., Da Silva M.V., Hansen P.J. (2017). A single nucleotide polymorphism in COQ9 affects mitochondrial and ovarian function and fertility in Holstein cows. Biol. Reprod..

